# To What Extent the Emergency Physicians in Riyadh City Are Aware of Patient Radiation Exposure From Diagnostic Imaging Requested in the Emergency Department

**DOI:** 10.7759/cureus.8623

**Published:** 2020-06-15

**Authors:** Abdulrahman Y Alhawas, Yasser Alaska, Khaled Almohaimede, Musab Hamoud Almushayqih, Bayan Khalid Altassan, Majid Homiedan

**Affiliations:** 1 Radiology and Medical Imaging, College of Medicine, King Saud University, Riyadh, SAU; 2 Emergency Medicine, College of Medicine, King Saud University, Riyadh, SAU; 3 Radiology, College of Medicine, King Saud University, Riyadh, SAU; 4 Radiology, King Faisal Specialist Hospital and Research Center, Riyadh, SAU; 5 Diagnostic Radiology, College of Medicine, King Saud University, Riyadh, SAU

**Keywords:** radiation exposure, awareness, doctors, radiological investigations

## Abstract

Introduction

Radiological imaging that uses ionizing radiation in emergency departments (EDs) has increased with advances in radiological diagnostic methods. Emergency (ER) physicians’ awareness of the radiation doses and the associated cancer risks that the patients are exposed to was surveyed using a questionnaire.

Aims

To assess the ER physicians’ awareness of radiation doses associated with the diagnostic imaging and to describe their practice about discussing radiation risk with patients at different hospitals in Riyadh city, Saudi Arabia.

Methods

A prospective, questionnaire-based observational study was conducted in 2016 among 176 ER physicians at different hospitals in Riyadh city. The percentage knowledge score and the frequency of discussing radiation risk with patients based on responses to three scenarios were rated on a visual analog scale (VAS), where a score of 100 indicated that physicians would always discuss it.

Results

The overall mean knowledge score was 28% (95% CI: 22-34). None of the studied parameters (gender, experience, country of medical qualification, type of degree, and employment level) showed a significant correlation with the overall awareness of ED physicians about radiation exposure. Over three-quarters of physicians (77%) underestimated the lifetime risk of fatal cancer attributed to a single computed tomography (CT) scan of the abdomen. Majority of physicians (60%) reported never discussing radiation risk with patients. The frequency at which physicians were discussing radiation risk with patients varied greatly depending on the clinical scenario (mean VAS scores between 46 and 82).

Conclusions

ER physicians of different hospitals in Riyadh city had a varied knowledge about the radiation exposure risks, although overall the perception was inadequate. The physicians should receive education, and the diagnostic imaging request may need to include information on radiation doses and risks.

## Introduction

The acute care of any patient presenting to the emergency department (ED) involves rapid diagnosis and management of life-threatening injuries/conditions. Radiographic studies, including X-rays, computed tomography (CT), and other imaging studies, are frequently used in the initial evaluation of the patient presenting to an emergency to delineate and define the disease process as well as to detect injuries that may be occult [1]. The patient was exposed to ionizing radiation, which was associated with the long-term development of cancer, even at low doses [2].

The accessibility and enhancements in diagnostic imaging techniques have led to a seven-fold increase in the use of radiological imaging modalities [3]. This is especially true for CT, which imparts more than 50% of all radiation exposure from diagnostic imaging [4]. Studies reported a 7.8% annual increase in the use of CT from 1996 to 2010, representing an overall doubling of the mean per capita effective dose of ionizing radiation [5].

In particular, injured or trauma patients are at risk of high-dose radiation exposure. Trauma patients often receive multiple CT scans and radiographs during their hospitalization [6]. Trauma patients are also more susceptible to the effects of radiation because they tend to be young [7]. Radiation exposure is associated with the development of cancer [8], and the young are much more exposed to these effects than the elderly.

The radiation dose given in any diagnostic imaging must be able to answer the clinical question at hand, with minimum possible risk to the patient [9]. Modern imaging equipment is advanced enough to make adjustments according to the size and anatomy of a particular patient [10]. This is important for the fact that the lifetime attributable risk of fatal cancer in children exposed to radiation is considerably higher than that of adults [11]. Therefore, it is important that doctors who are requesting these imaging modalities should know its associated risks to the patients. The time-pressured environment in an ED, where many radiological imaging tests are asked daily, places more emphasis on knowing the indications and risks of the imaging techniques [12]. In Saudi Arabia, there are not enough data collected yet on the emergency (ER) physicians’ perception of the risks associated with patient radiation exposure. Thus, this study aims to assess the level of awareness of ER physicians regarding the knowledge of imaging radiation doses and related risks.

## Materials and methods

A cross-sectional study was performed in the EDs of different hospitals in Riyadh city, Saudi Arabia, using a convenient sampling technique. The majority of participants were from King Khalid University Hospital, whereas the rest were from the following hospitals: King Abdulaziz University Hospital, National Guard Hospital, Security Forces Hospital, King Faisal Specialist Hospital, King Saud Medical City Hospital, Prince Sultan Military Medical City, and King Fahad Medical City.

A previously validated adopted self-administered questionnaire [12] in English language was transformed into an electronic version and sent to the participants to collect the data of our study, which comprised three sections. The first section covered demographic data and also included questions about receiving formal education in radiation exposure risks. The second one involved three common clinical scenarios aimed to investigate how frequently doctors would discuss with their patients about the risks of radiation. The last part was built to measure the participants’ knowledge of radiation exposure levels using 15-item multiple-choice questions that encompassed the concept of lifetime risk, background environmental radiation, and effective dose of different imaging modalities.

Statistical analysis

Data were analyzed using SPSS Version 22.0 (IBM Corp., Armonk, NY, USA). A p-value of 0.05 or less was considered significant.

Ethical consideration

Participant's anonymity was assured by assigning each participant with a code number for analysis only. The respondents were given a brief description of the study and its objectives.

## Results

In this study, an electronic survey was sent to 176 doctors, of whom 159 completed it, giving a response rate of 90%. No more than 2% of data were missing for any variable. Respondents were mostly men (81%), working for more than three years (69%) and had their medical degree from Saudi Arabia (87.5%), as shown in Table 1.

**Table 1:** Demographic characteristics of participating clinicians *Whether MBBS or equivalent was undertaken and completed as an undergraduate or postgraduate student.

Demographic characteristics	Number	Percent
Gender		
Male	143	81
Female	33	19
Experience level		
≤3 years	54	30.7
>3 years	122	69.3
Country of medical degree		
Saudi Arabia	154	87.5
Arab countries	16	9.1
Others	06	3.4
Type of degree^*^		
Undergraduate	119	67.6
Postgraduate	57	32.4
Current employment level		
Consultant	63	35.8
Senior registrar	20	11.4
Registrar/resident/fellow	62	35.2
Intern	31	17.6

Our results indicated that the majority (60%) of doctors reported never having had any formal training on risks to patients from radiation exposure. More than one-third would like to receive formal training on risks and doses of radiation exposure from common radiological investigations. The mean knowledge level for all physicians was 28% (95% CI: 22-34). Physicians who had received formal training scored the same as those who had not (28%). Female doctors scored less (23%) as compared to their male colleagues (28%), but this was not statistically significant (p= 0.681) possibly due to wide confidence intervals. There was a statistically significant difference among visual analog scale (VAS) scores for various clinical scenarios and studied parameters, as shown in Table 2.

**Table 2:** Scores of radiation knowledge and VAS* according to three clinical scenarios VAS: A scale from 0 to 10, where  0 denotes never and 10 indicates always. *Statistically significant at a p-value of ≤0.05. VAS, visual analog scale

Characteristic	Radiation knowledge score	Clinical scenario mean VAS score		
		1	2	3
Total (n=176)	28 (22–34)	82 (77–86)	79 (73–84)	46 (41–52)
Gender				
Male, n=143 (81%)	28 (24–33)	81 (76–86)	77 (71–83)	45 (38–51)*
Female, n=33 (19%)	23 (17–29)	82 (72–93)	87 (76–98)	56 (43–69)
Experience level				
<3 years, n=54 (30.7%)	27 (22–33)	72 (62–81)	73 (62–83)	46 (37–56)
>3 years, n=122 (69.3%)	28 (22–34)	85 (80–90)*	81 (75–87)	47 (40–54)
Country of a medical degree				
Saudi Arabia, n=154 (87.5%)	28 (21–34)	81 (76–86)	79 (73–85)	47 (41–53)
Arab countries, n=16 (9.1%)	27 (20–35)	82 (65–99)	74 (54–94)	44 (24–64)
Others, n=06 (3.4%)	30 (24–35)	93 (75–110)	85 (57–113)	42 (10–75)
Type of degree				
Undergraduate, n=119 (67.6%)	29 (24–34)	84 (79–90)*	81 (75–88)	44 (37–51)
Postgraduate, n=57 (32.4%)	28 (20–35)	75 (65–84)	73 (63–84)	52 (42–62)
Current employment level				
Consultant, n=63 (35.8%)	27 (23–32)	85 (78–93)	76 (66–86)	46 (35–57)
Senior registrar, n=20 (11.4%)	30 (24–35)	92 (82–103)	94 (87–101)*	49 (31–67)
Registrar/fellow/resident, n=62 (35.2%)	26 (19–34)	76 (67–85)	77 (68–87)	42 (33–51)
Intern, n=31 (17.6%)	26 (18–35)	74 (61–87)	72 (56–89)	58 (45–71)

Our results found that more than two-thirds of the doctors underestimated the radiation exposure from lumbar spine X-ray, and around half of the doctors underestimated the radiation exposure for seven other common radiological investigations, as shown in Table 3.

**Table 3:** Overall results of participants’ responses to the 15-item radiation knowledge component of the questionnaire CXR, chest X-ray

	Underestimated	Correct	Overestimated
Limb X-ray (0–1 CXRs)	0	97 (55.1%)	79 (44.9%)
Lumbar spine X-ray (50–100 CXRs)	132 (75%)	21 (11.9%)	23 (13.1%)
CXR (0–1 CXRs)	0	88 (50%)	88 (50%)
Upper GIT X-ray (10–50 CXRs)	96 (54.6%)	37 (21%)	43 (24.4%)
Lower GIT X-ray (10–50 CXRs)	89 (50.6%)	38 (21.6%)	49 (27.8%)
CT scan of the abdomen (100–500 CXRs)	89 (50.6%)	38 (21.6%)	49 (27.8%)
Ultrasound of the abdomen (0–1 CXRs)	0	123 (69.9%)	53 (30.1%)
CT scan of the head (50–100 CXRs)	90 (51.1%)	36 (20.5%)	50 (28.4%)
MRI of the head (0–1 CXRs)	0	123 (69.9%)	53 (30.1%)
CT scan of the chest (100–500 CXRs)	103 (58.5%)	33 (18.8%)	40 (22.7%)
The lifetime risk of fatal cancer from a single CT scan of the abdomen (1 in 2,000)	135 (76.7%)	33 (18.8%)	8 (4.5%)
Days of background environmental radiation equivalent to a single CXR (3)	14 (8%)	26 (14.8%)	136 (77.3%)
No. of CXRs equating to radiation exposure on a 20-hour flight from Riyadh to Los Angeles (5)	90 (51.1%)	30 (17%)	56 (31.8%)
Radiation absorbed of a single CXR (0.01 mSv)	39 (22.2%)	55 (31.3%)	82 (46.6%)

## Discussion

The excessive use of medical radiological investigations is a significant cause of the increasing radiation exposure of the general population. Consequently, radiation protection is a topic of considerable scientific concern. Our study showed that doctors’ knowledge of radiation exposure from radiological investigations is unsatisfactory. The mean knowledge score for all participant physicians was 28%. It seems to be low in comparison to another study in Australia (40%) [12]. Overall, ER doctors underestimated the radiation exposure of routine radiological investigations and the associated risks. Ignorance of actual doses and risks of radiation is the primary cause of frequent use of diagnostic imaging techniques.

This lack of knowledge may be because of various factors. A significant burden of this lack of knowledge can be attributed to the education provided to our physicians at various levels, with a majority of the doctors denying having any previous academic knowledge regarding radiation hazards. Previous surveys also showed low-to-moderate knowledge among physicians concerning radiation doses and the relevant risks [12,13]. Various studies have proved that doctors having formal training about ionizing radiation performed much better than those with no previous training [14]. However, other studies reported no difference in knowledge of physicians who attended radiation safety courses and those who did not [12,15].

Our study has clearly shown that awareness of radiation hazard from diagnostic imaging lacks among residents and interns, whereas the senior medical staff performed significantly better. However, similar differences in radiation knowledge were found among subgroups of respondents in other researches [12,14]. Also, our study indicated that formal training increases the physician's awareness about radiation hazards, similar to previous studies [14,16].

Our survey confirmed that the physician's awareness of radiation doses from standard radiological procedures is inadequate. Many previous studies also indicated that overall awareness of this area is poor and that doctors often underestimate the radiation dose [14,17]. Furthermore, physicians’ choice of patient counseling regarding radiation hazards is highly conditional. One study showed that only 7% of patients who were subjected to abdominal CT scan were given information on radiation exposure [18], whereas another study including a 6-year-old with a minor head injury revealed that physicians would often discuss the risks with the parents [12]. This indicates the need to educate physicians about ionizing radiation relevant to diagnostic imaging and their clinical role to discuss radiation exposure risks with their patients.

We performed a cross-sectional questionnaire from 176 ER physicians, none of who knew the approximate dose of radiation received to a patient during a chest X-ray or even the measurement in units of radiation (0.02 mSv). The estimated doses of radiation exposure were much lower than the correct ones. This indicates that doctors were exposing patients to a radiation dose that was much higher than expectations. More than one-half of the respondents underestimated the radiation dose from commonly requested radiological procedures similar to that reported in other studies [16,17].

In our study, the respondents (53%) incorrectly believed that ultrasound and MRI emit ionizing radiations. While other studies revealed a percentage of no more than 28% of respondents [12,16], this shows to be a defect in the principle knowledge of diagnostic radiology. Accordingly, the clinical justification for each radiological investigation should be given relevant to the radiation risk and possible diagnostic benefits [19].

In the current results, despite the general underestimation of radiation exposure, the actual dose (in mSv) of chest X-ray was overestimated by 46% of doctors. This shows the physicians unfamiliarity with all units of radiation. Surprisingly, 50% of doctors responded that a chest X-ray was equivalent to more than one chest X-ray. Our results are in agreement with an Australian research [12].

Majority (76%) of our participants underestimated the risk of cancer development after radiation exposure of radiological investigations. Similarly, other researches also showed that doctors underestimate the risk of cancer development after radiation exposure from radiological investigations [11,13]. The attributable cancer risk from single radiation exposure is small. However, the cumulative effects of multiple exposures overtime should be kept in mind [20,21]. We aim to promote the safe use of medical imaging devices and increase patient awareness of their exposure.

Our study had various limitations, including the fact that it shows only a small percentage of female ER physicians. However, there was no selection bias, as we examined all eligible doctors, with a high response rate, that showed the sample was a true representative. Our results may be biased, as all published studies use variable questions. Also, there are marked differences in the health care systems between the various countries, which makes the overall conclusion using a common standard difficult. Still, this study shows that radiation protection awareness among physicians should be improved.

It was suggested that radiation doses and related risks should be provided on imaging request forms [22]. That is how doctors can consider this information better and discuss the risks with the patient, consequently increasing doctors’ general awareness and knowledge.

## Conclusions

ER physicians of different hospitals in Riyadh city demonstrated a varied knowledge of the risks from radiation exposure, but overall knowledge was inadequate. ER physicians should receive formal education and training, and the diagnostic imaging request process may require information on radiation doses and risks as a second layer to ensure that requesting clinicians get hands-on knowledge about the radiation dose for commonly requested radiological investigations.

## Figures and Tables

**Figure 1:**
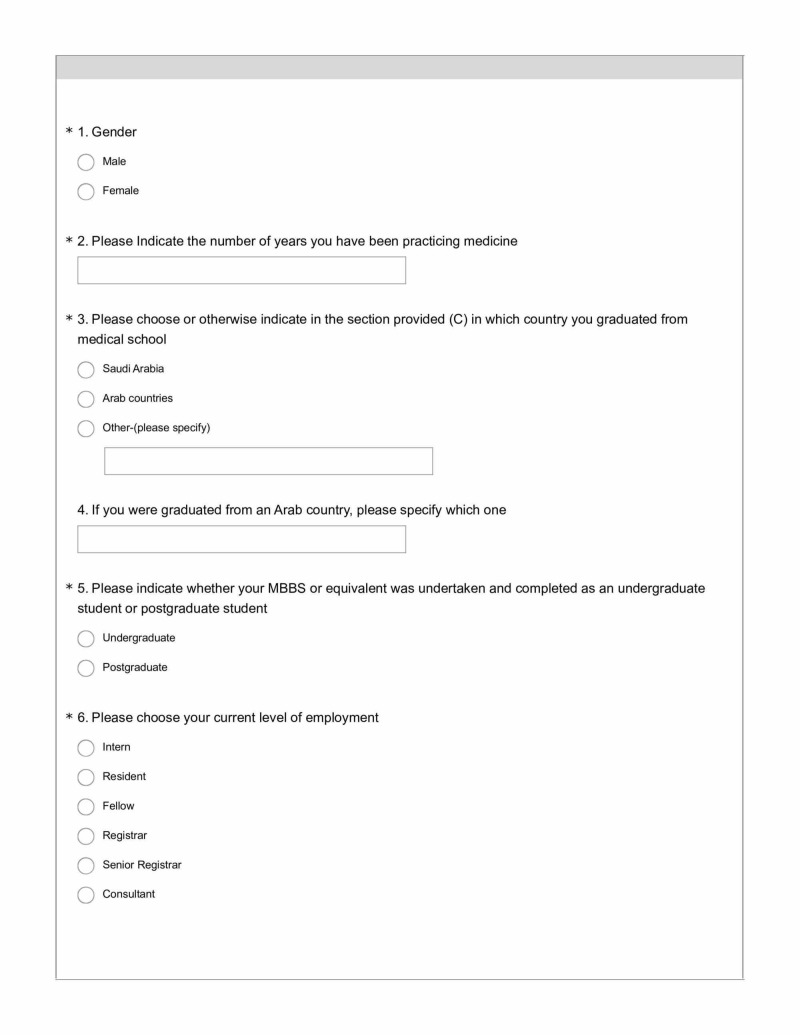
Questionnaire

**Figure 2:**
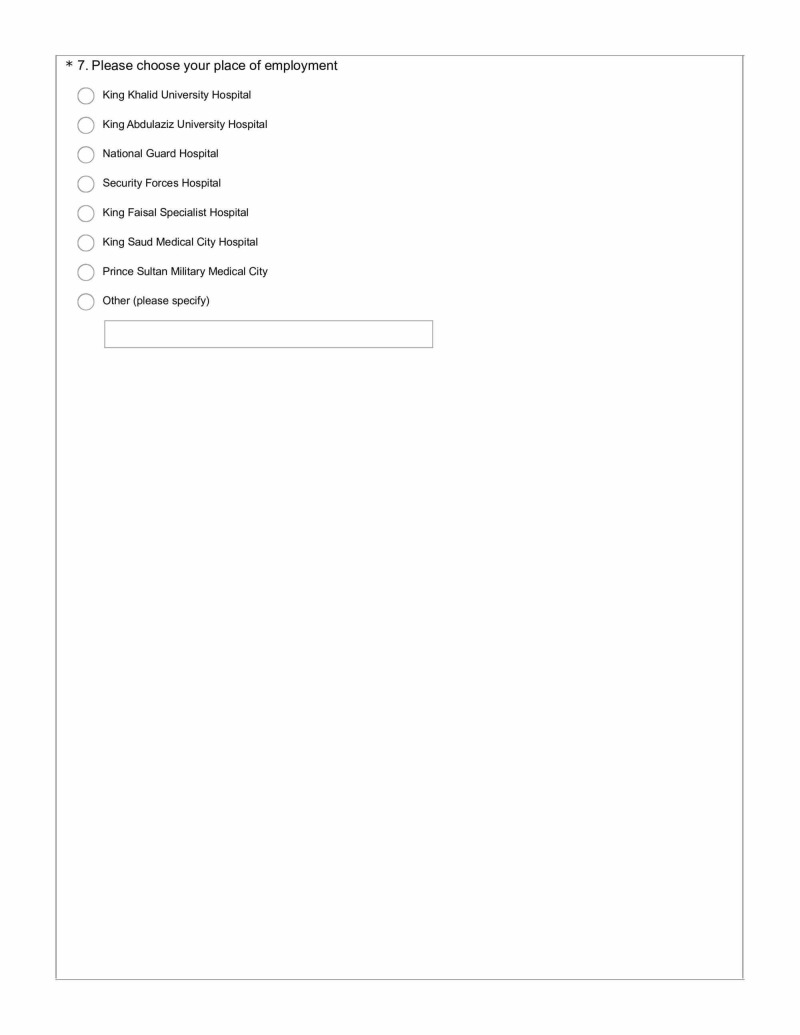
Questionnaire

**Figure 3:**
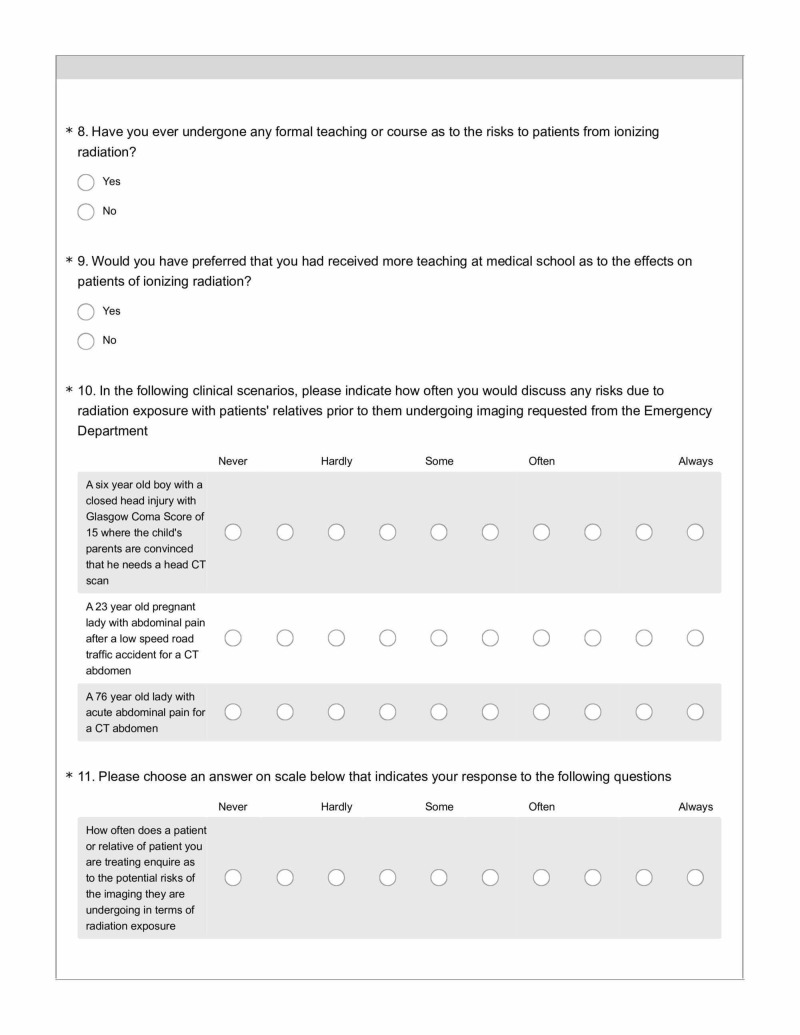
Questionnaire

**Figure 4:**
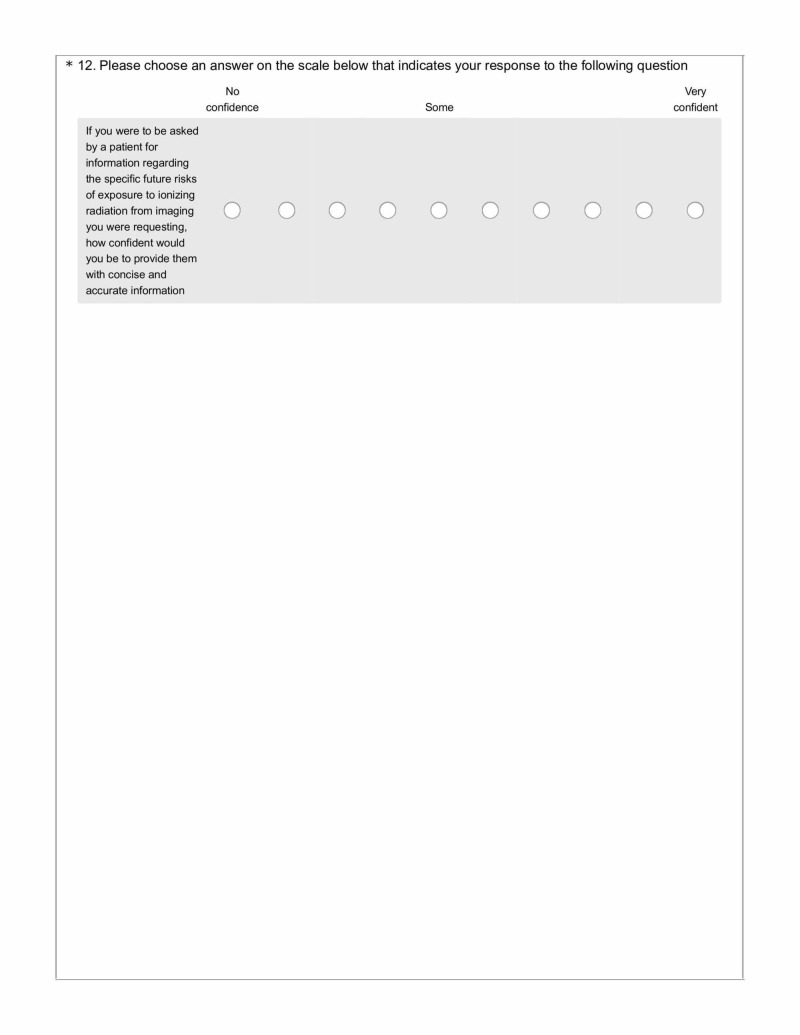
Questionnaire

**Figure 5:**
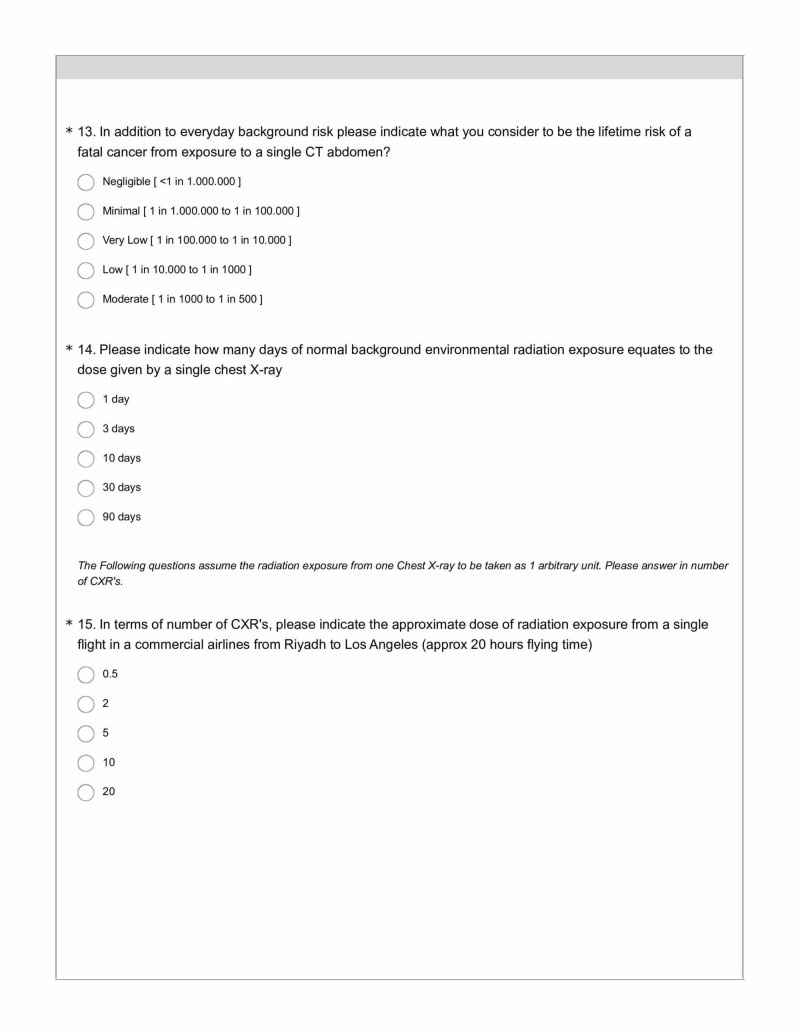
Questionnaire

## References

[1] Beatty L, Furey E, Daniels C, Berman A, Tallon JM (2015). Radiation exposure from CT scanning in the resuscitative phase of trauma care: a level one trauma centre experience. CJEM.

[2] Nickoloff EL, Alderson PO (2001). Radiation exposures to patients from CT: reality, public perception, and policy. AJR Am J Roentgenol.

[3] Committee on Health Risks of Exposure to Low Levels of Ionizing Radiations (2006). Health Risks from Exposure to Low Levels of Ionizing Radiation: BEIR VII Phase 2 (2006).

[4] Schauer DA, Linton OW (2009). NCRP Report No. 160, Ionizing Radiation Exposure of the Population of the United States, medical exposure--are we doing less with more, and is there a role for health physicists?. Health Phys.

[5] Smith-Bindman R, Miglioretti DL, Johnson E (2012). Use of diagnostic imaging studies and associated radiation exposure for patients enrolled in large integrated health care systems, 1996-2010. JAMA.

[6] Kim PK, Gracias VH, Maidment AD, O'Shea M, Reilly PM, Schwab CW (2004). Cumulative radiation dose caused by radiologic studies in critically ill trauma patients. J Trauma.

[7] Mackenzie E, Fowler C (2003). Epidemiology of injury. Trauma.

[8] Shimizu Y, Schull WJ, Kato H (1990). Cancer risk among atomic bomb survivors. The RERF Life Span Study. Radiation Effects Research Foundation. JAMA.

[9] Cember H, Johnson TE (1996). Introduction to Health Physics.

[10] Paterson A, Frush DP, Donnelly LF (2001). Helical CT of the body: are settings adjusted for pediatric patients?. AJR Am J Roentgenol.

[11] Brenner DJ, Elliston CD, Hall EJ, Berdon WE (2001). Estimated risks of radiation-induced fatal cancer from pediatric CT. AJR Am J Roentgenol.

[12] Keijzers GB, Britton CJ (2010). Doctors' knowledge of patient radiation exposure from diagnostic imaging requested in the emergency department. Med J Aust.

[13] Krille L, Hammer GP, Merzenich H, Zeeb H (2010). Systematic review on physician's knowledge about radiation doses and radiation risks of computed tomography. Eur J Radiol.

[14] Soye JA, Paterson A (2008). A survey of awareness of radiation dose among health professionals in Northern Ireland. Br J Radiol.

[15] Quinn AD, Taylor CG, Sabharwal T, Sikdar T (1997). Radiation protection awareness in non-radiologists. Br J Radiol.

[16] Zhou GZ, Wong DD, Nguyen LK, Mendelson RM (2010). Student and intern awareness of ionising radiation exposure from common diagnostic imaging procedures. J Med Imaging Radiat Oncol.

[17] Shiralkar S, Rennie A, Snow M, Galland RB, Lewis MH, Gower-Thomas K (2003). Doctors' knowledge of radiation exposure: questionnaire study. BMJ.

[18] Lee CI, Haims AH, Monico EP, Brink JA, Forman HP (2004). Diagnostic CT scans: assessment of patient, physician, and radiologist awareness of radiation dose and possible risks. Radiology.

[19] Street M, Brady Z, Every B, Thomson K (2009). Radiation exposure and the justification of computed tomography scanning in an Australian hospital emergency department. Intern Med J.

[20] Lavoipierre AM (2011). Doctors' knowledge of patient radiation exposure from diagnostic imaging requested in the emergency department. Med J Aust.

[21] Nguyen PK, Wu JC (2011). Radiation exposure from imaging tests: is there an increased cancer risk?. Expert Rev Cardiovasc Ther.

[22] Grove ML (2003). Doctors' knowledge of exposure to ionising radiation: just tell them the dose. BMJ.

